# Design and Implementation of a Low-Energy-Consumption Air-Conditioning Control System for Smart Vehicle

**DOI:** 10.1155/2019/3858560

**Published:** 2019-08-27

**Authors:** Chien-Lun Weng, Lih-Jen Kau

**Affiliations:** Department of Electronic Engineering, National Taipei University of Technology, No. 1, Sec. 3, Chung-Hsiao E. Rd., Taipei 10608, Taiwan

## Abstract

About 7% of people's daily time is spent in taking vehicles between office and home. Besides, with the improvement of the living standard in today's society, people's requirements for a comfortable environment inside the car are constantly increasing and this must rely on an effective vehicle air conditioner to maintain the comfort of the cabin environment. In general, a vehicle air conditioner uses the air-mixing mode to regulate the temperature control system. In this mode of operation, the compressor needs to work continuously, which is extremely energy consuming. The vehicle's air conditioner is greatly affected by the inner and outer heat load, which are generated therein. Furthermore, the heat load is instantly changeable. Therefore, only when the controller can adapt to the feature of heat load, then we can find the optimal control method, thus enabling the vehicle's air conditioner to interact with the actual heat load to supply the balanced cooling capacity and, as a result, create the most comfortable environment inside the cabin with minimum energy consumption. For this purpose, we bring up in this paper a low-energy-consumption smart vehicle air-conditioning control system to detect total heat load, which can change the vehicle's air-conditioning capacity mode to maintain the average temperature at 25.2°C∼26.2°C and the average humidity at 46.6%∼54.4% in the cabin. When the inner heat load is stable, the rest times of the compressor can reach 16∼23 times per hour, which attains a rate of fuel saving around 21%∼28%. With the proposed architecture, the purpose of the low-energy-consumption vehicle air-conditioning system can be achieved, which, at the same time, creates a comfortable environment inside the cabin.

## 1. Introduction

While people are pursuing both economic and technologic development, massive fossil fuels are burnt, which has almost used up the energy that have been accumulated for centuries and is destroying the environment, making the living condition on Earth deteriorate rapidly. Technologic development results in the air pollution, such as smog, PM_2.5_ and CO_2_, etc.; among them, traffic exhaust emission contributes about 30∼40% of the total air pollutants. In Taiwan, the traffic exhaust emission shares up to 37% of total air pollutants [1]. These emissions incur global warming, climate change, and formation of extreme climate, which are the main reasons causing the meteorological disaster to spread over the entire world.

These phenomena force people to focus on the environmental awareness; countries all over the world have set up environmental organizations and signed conventions to prevent human beings from continuing to undermine the Earth's ecological environment. COP21, which was the first global agreement in history, used to prevent climate change, and officially valid since November 4, 2016. In order to drive the COP21 policy, all governments worldwide are committed to developing vehicles with low energy consumption, low carbon emissions, and low pollution to slow down the speed of environmental pollution and destruction. In addition, since the vehicle air-conditioning has been the standard equipment on all modern vehicles, it provides a comfortable in-car environment for driving and passengers; however, the massive energy consumed by car air conditioners is the major source of vehicle pollution.

Although in-car space is not big, yet, due to the reason that car is long time exposed in natural environment for driving, the vehicle air-conditioning heat load has the instant change feature; the range between maximum and minimum heat load is tremendously large. At present, the vehicle air-conditioning system is limited by engine rpm, and the refrigerating capacity becomes a variable, resulting in unbalance between in-car heat loads and refrigerating capacity. At present, the thermostatic control on vehicle air-conditioning system makes adjustment by mixing the warm and cold air, which is extremely energy consuming; in addition, the in-car relative humidity (RH) is very low and thus makes in-car passengers dehydrate and easily get tired; also, it increases the car's energy consumption. In order to improve this defect, the key point is to adjust vehicle air-conditioning control system of massive vehicles that consume massive energy and produce pollution and enable the vehicle A/C to make optima operation. Except providing comfort to in-car passengers, it will also elevate the energy-consuming efficiency and mitigate damage to Earth's ecology, as well as contributing to build the sustainable Earth.

### 1.1. Main Factors That Affect the Comfort of Human Beings

“Comfortable environment” is an ideal life style that human beings have been long pursuing. After the Industrial Revolution, human life quality has been upgrading rapidly. In order to improve in-door environment and comfort, it also has polluted the outer environment in return. With the rapid and prosperous scientific/technologic development, urban traffics become more convenient; people use transporting tools to their destinations. Research shows that people spend about 7% of their daily time on boarding vehicles [2]; therefore, in-car comfort becomes extremely important.

The main factors affecting human being's comfort are indoor temperature, relative humidity, wind speed, noise, etc. According to the ASHRAE 55-2013 Specification [3], the metabolic rate of sitting human body is 1.2 Met (1 Met = 58.15 W/m^2^); under typical clothing condition, the summer comfort temperature in Taiwan is 23°C∼26°C and winter is 18°C∼20°C. Fluctuation of relative humidity at high or low temperatures becomes very important to human body's heat balance and thermal sense [4]. In Taiwan, we feel comfortable at 50%∼60% RH. Airflow distribution is within 0.15∼0.3 (m/sec) per minute. Strong air flow will cause discomfort easily; the more gentle air flow performed in the indoor air-conditioning environment, the more comfortable human body feel can achieve.

### 1.2. Inner/Outer Environment Status of Current Vehicle

#### 1.2.1. Out-of-Car Environment

Air pollution is caused not only by the pollutants emitted from factories and vehicles, but dust storms from the Gobi Desert, plant's dust, volatile organic compounds (VOCs), and photochemical smog reactions are all part of air pollution as well [5].

Gases exhausted from traffic tools contain the hazardous particulate matters (PM), SO_2_, NO_X_, CO, and ozone (O_3_) [5–7], closely related to the human respiratory system and cardiovascular disease. Long-term or short-term exposure in the PM (PM_10_, PM_2.5_) environment will induce cardiac contractility and decreased vascular elasticity, resulting in inadequate perfusion, which may be an important factor in causing cardiovascular system damage [8]. Prolonged exposure to environmental air pollution will easily cause allergic reaction of the respiratory system, the most common symptom being asthma, one of the most common chronic diseases in children, mainly caused by the over-reaction of trachea that leads to contractions [9–11].

#### 1.2.2. In-Car Environment

The scientific/technologic development and environmental protection awareness are rising up, and people's requirements on living quality are upgrading too. The air-conditioning system is a necessity in modern life because it provides a comfortable activity environment and meets people's most basic physical needs. From the survey report, Americans spend about 89% of their daily time indoors and the remaining 11% are on transporting vehicles and outdoors [12]. The degree of environmental comfort is greatly influential to people's work efficiency, health, and environment.

Related indoor air quality research is not restricted to buildings; it also surveys semiclosed transporting vehicles. Studies have found that everyday, people spend much time on their way between offices and homes by taking transporting tools; approximately 7% of the daily time is spent on taking transporting tools [2].

Using vehicle air conditioning makes drivers and passengers feel comfortable in the car and is an important indicator of in-car comfort [13]. When starting the vehicle air conditioning, it cools down and dehumidifies in-car air, bringing the in-car environment into a low-temperature and low-humidity state. When a person is in this environment, the body will spread heat loss to the environment through conduction and radiate, in which 70% of body heat loss is caused by conduction and radiate, and 27% is evaporated by sweat from the skin [14]. Therefore, staying in the air-conditioned environment for a long time will easily cause water loss from human body. During long driving, both driver and passengers will feel tired [15].

### 1.3. Variation of Vehicle Air-Conditioning Load

Nowadays, our living level is elevated. In Taiwan, each family has at least 1∼2 vehicles; to sustain the in-car comfort, it must rely on the car's air-conditioning system. In-car space is not big, but during the driving, the car exposes to sunlight and in-car heat is generated, therein causing extremely large heat load. The maximum heat load can be 10 times over the minimum one. For conventional internal combustion engine, the vehicle air-conditioning compressor is driven by engine crankshaft and the cooling capacity is restricted to engine speed and is proportional to engine rpm; the ratio of max/min output capacity is over 3 times [16]. Although the air-conditioning system of electric vehicle runs independently and since it is limited to the battery jar capacity, it has to reduce the energy consumption from the air-conditioning system to elevate car's durability [17–21]. Thus, the fit of vehicle air-conditioning demand and energy supply is extremely important; when the car is starting up or at low-speed state, in-car space temperature may rise up to 60°C due to being exposed to sunlight; yet, output of car air-conditioning refrigerating capacity is low due to low engine speed, and the heat load input/output is unbalanced, which will make in-car passengers feel too warm and uncomfortable [16]. After the car drives for a while, in-car temperature reduces gradually and heat load is down. Since the car is at high-speed state, supply of refrigerating capacity is high and too much cool air is supplied and passengers feel too cold and uncomfortable again. Since normally the vehicle air-conditioning system mixes warm and cold air to adjust the cooling state, which makes in-car temperature approaches the setting one, driver and passengers can feel comfortable.

This unbalanced air-conditioning system is not only unable to provide comfort to people, when in-car passengers feel comfortable by means of mixing of warm and cold air to regulate air temperature, the system will also dehumidify the in-car air that reduces the relative humidity (RH) to as low as below 40% too. It thus makes in-car passengers dehydrate and easily get tired and increases the driving danger. In addition, it is an extremely energy-consuming air-conditioning control method.

Heat sources that affect vehicle's heat load are divided into two categories:

  In-car generated heat load: this includes heat coming from the engine and human body.

  Heat load coming from the outer ambient atmosphere: heat load coming from car top, side doors, chassis, and glass windows (heat of radiation, approx. 30% of it will become in-car heat load) and warm wind from outer ambient environment.

### 1.4. Improve Vehicle Air-Conditioning's Comfort Effect by Low Energy Consumption

Vehicle air conditioning now has become the standard equipment of modern vehicles. It can provide drivers and passengers a comfortable in-car environment. Yet, turning on the air-conditioning system will increase engine loading and energy loss. Factors that affect vehicle's energy consumption are the driving habit, weather status, traffic status, vehicle status, etc. Among them, the one influencing the oil consumption most is initiating the vehicle air conditioning, and average oil consumption will increase up to 21% from it [22]; for an electric vehicle, after initiating the air-conditioning system or electric-heating system, the durability drops over 50% obviously. Therefore, developing a low-energy-consumption air-conditioning system becomes an important indicator in future vehicle development.

Nowadays, the energy-saving control mode of vehicle air conditioners can be achieved by establishing a prediction model for the number of passengers and the ambient temperature [17] or with the battery cooling and motor waste heat recovery for the management of temperature [18, 19]. Air-conditioning performance can also be improved through refrigerant improvement [20, 21]. For both electric vehicles and hybrid electric vehicles, the air conditioner uses the air-mixing mode to regulate the temperature control system. However, in this mode of operation, the compressor needs to work continuously, which is extremely energy consuming. Moreover, the vehicle air-conditioning matter is against both inner and outer big heat loads, which have “instantly change” feature. Thus, if we can adapt to the load characteristics and find out the optimal control method and enable vehicle A/C to supply cold energy per actual heat load required, then we can achieve a balance between supply and demand and create the most comfortable in-car environment with the lowest energy consumption.

Motivation of performing this study is to seek the optima control method of the vehicle air-conditioning system. Regarding the “instantly change” feature in vehicle air-conditioning load, we studied to eliminate the existed defects that include too low temperature/humidity and high oil consumption. The main purpose is to follow the heat load characteristics and adjust/control the air-conditioning capacity per load demand, create the optimal energy-saving operation mode that supplies the exactly required in-car cooling capacity, stably maintain the in-car most comfortable environment with low-energy consumption, and effectively reduce the environmental pollution and destruction.

This article's structure is illustrated as follows: Section 2 illustrates the innovative low-energy-consumption vehicle air-conditioning control system structure; Section 3 performs tests and comparison on the experimental configuration and performance of designed innovative system; and Section 4 gives the conclusion.

## 2. Structure of Low-Energy-Consumption Vehicle Air-Conditioning Control System

As illustrated above, the purpose of this study is to achieve the optima control of low-energy-consumption vehicle air-conditioning system, interpret the structure and features of conventional vehicle air-conditioning system and bring up the structure of low-energy-consumption vehicle air-conditioning control system studied to improve the defects and problems on the conventional system. In this section, we are interpreting the structure of the planned system in detail.

### 2.1. Structure of Vehicle Air-Conditioning System

**Figure 1:**
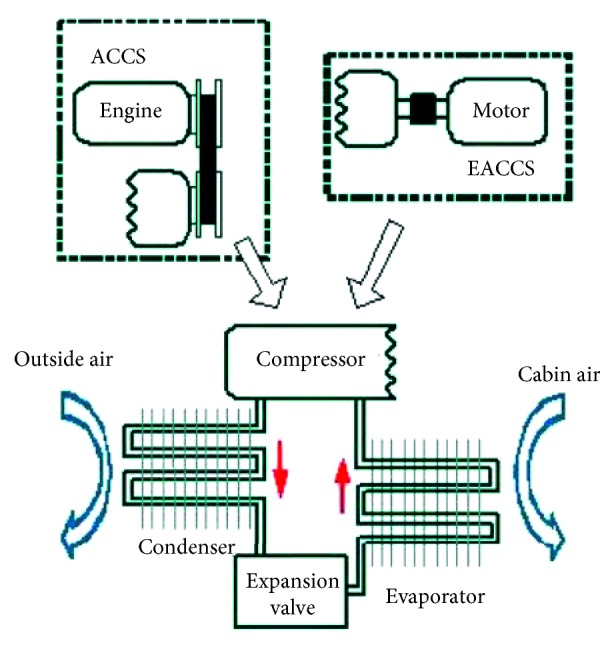
Structure figure of air-conditioning circulation in conventional internal combustion engine and electric vehicle [23].

Along with the higher requirement of in-car comfort, the vehicle air-conditioning system can provide cooled, heated, dehumidified, and defogged air; ways to drive the vehicle air-conditioning system mainly are two: one is the pulley compressor system; the other one is the electric compressor system, shown in Figure 1 [23].

**Figure 2:**
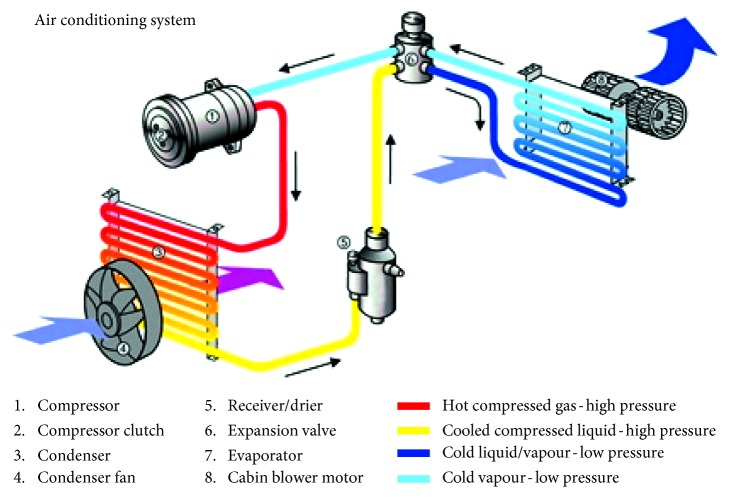
Vehicle air conditioning system [24].

**Figure 3:**
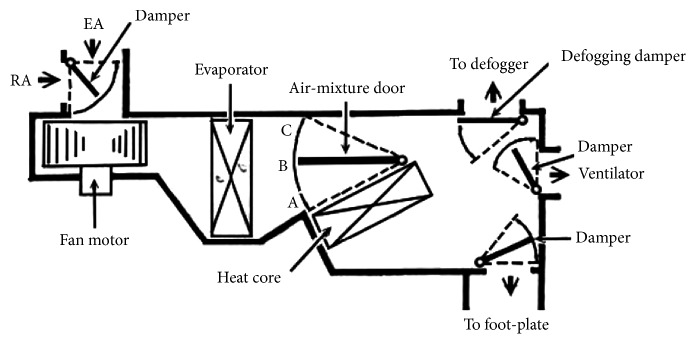
Structure of air-mixing box in vehicle air-conditioning system [16].

The vehicle air-conditioning structure is shown in Figure 2 [24]; it is mainly configured by using a compressor, condenser, evaporator, and four-way valve. Among them, the most critical one is the compressor. The other important structure is the air-mixing box that mixes both warm and cold air, as shown in Figure 3 [16]. At present, most vehicle air-conditioning equipment applies air-mixing way to control in-car temperature effectively. Yet, since vehicle air-conditioning compressor connects to car engine by transmission belt, it normally works at peak capacity, which not only shortens part's operating lifetime but also wastes unnecessary energy during the air-conditioning process.

#### 2.1.1. Pulley Compressor

At present, the vehicle air-conditioning system of fuel-oil vehicle is driven by internal combustion engine; the engine rotates to drive the compressor over belt pulley; after evaporator has absorbed in-car heat, heat is emitted to outer environment via condenser to form cooling effect. Heating effect is made over inducing engine's cooling water into the heat core in air-conditioning assembly and has the in-car air flow over the heat core to heat up and is sent to in-car space, as shown in Figure 3 [16]. To sustain in-car comfort, it has to mix both the cold and warm air to meet thermostatic effect; yet, the side effect is that the in-car air humidity is too low and energy consumption is heavy.

Air conditioning on fuel-oil vehicles works by using a engine-drive compressor, and its cooling effect is proportional to the compressor and engine rpm, which indirectly increases engine's load and makes vehicle increase its oil consumption while the A/C system is initiated. The advantages and disadvantages of pulley compressors in the vehicle air-conditioning system are as follows:

  Advantage: mature technologies, easy for thermostatic control; use engine's cooling water to provide in-car heated air; the heat source supply is stable.
  Disadvantage: thermostatic control has to make adjustment through mixing the warm/cold air; while at engine's idling speed state, the cooling/heating effects are not good. Since the cooling effect is proportional to engine output capacity, ratio of maximum and minimum output capacity is always over three times that of waste energy; vehicle cannot normally supply air-conditioning service while vehicle is stationary.

#### 2.1.2. Electric Compressor

With the rise of environmental awareness, vehicle technology focus gradually shifts from traditional internal combustion engine to electric motor. With this evolution, the technical development of the dynamic system and energy storage system of an air-conditioning system structure also changes. As shown in Figure 1 [23], the electric compressor that integrates the drive motor, compressor, and drive circuit has replaced the conventional pulley compressor, which enables the air-conditioning system to work independently, and will not increase the load of dynamical system. The system is able to adjust the work status freely per requirement and avoid consuming additional power output; yet, it still is limited by battery jar capacity. Only reducing energy consumption of the air-conditioning system can elevate the durability of electric vehicle effectively [25].

Normally, the electric vehicle is equipped with devices that can provide stable high-temperature media; the way of using engine's hot cooling water to provide heated air is also replaced by electric heating, whereas the conversion of electric heating providing required in-car heated air is always limited by electric-heating conversion and heat conduction efficiency. In addition, the electric-heating system is an extremely high energy-consuming equipment that makes the durability of electric vehicle reduce over 50% severely. Advantages and disadvantages of the electric compressor air-conditioning system are as follows:

  Advantages: the air-conditioning system can run independently; compressor can run under frequency-varying control to elevate the overall air-conditioning efficiency; thermostatic control is easy.
  Disadvantages: the energy-consuming amount of the air-conditioning system affects the durability of electric vehicle. The electric-heating system is applied to supply heated air; the features of poor efficiency of energy conversion on the electric system and high energy consumption of electric-heating system make the durability of electric vehicle down obviously. In addition, the construction cost of the electric compressor and electric-heating system is relatively expensive.

The vehicle air-conditioning system provides a comfortable in-car environment to the driver and passengers; yet, from above, we know that the promotion of vehicle air-conditioning technology still has the energy waste problem while initiating the air-conditioning system on vehicle.

### 2.2. Structure of Planned Innovative Control System

**Figure 4:**
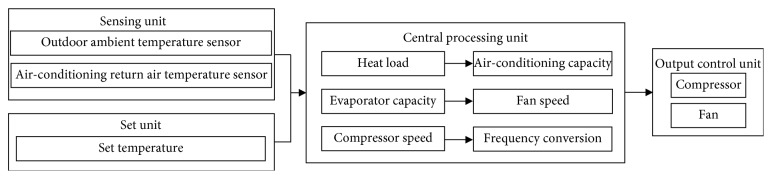
Structure diagram of low-energy-consumption smart vehicle air-conditioning control system.

At present, thermostatic control of the vehicle air-conditioning system mostly applies mixing of both warm and cold air and meets the goal of comforting in-car passengers with suitable temperature. This operation is very energy consuming; once the target temperature is met, it can also influenced the in-car air humidity and always result in low relative humidity condition. In this study, the structure of low-energy-consumption smart vehicle air-conditioning control system can be divided into the sensing unit, set unit, central processing unit (CPU), and output control unit, shown in Figure 4.

**Figure 5:**
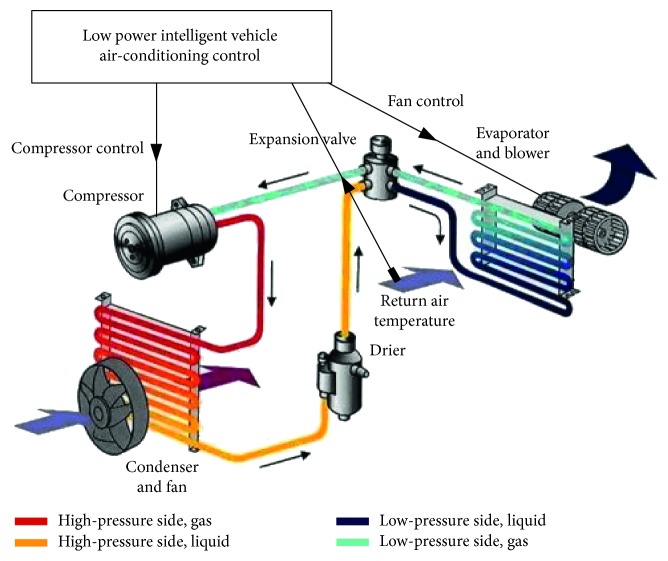
Low-energy-consumption smart vehicle air-conditioning system diagram.

**Figure 6:**
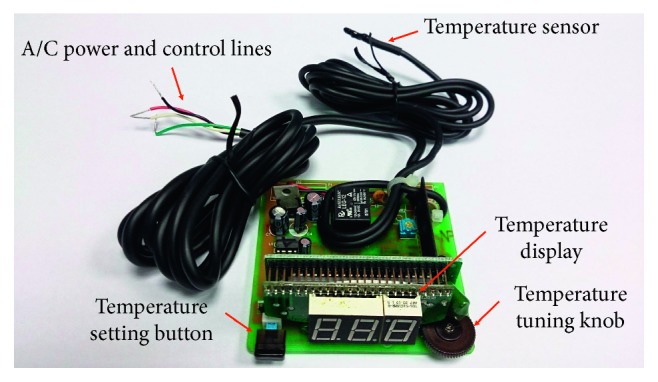
Solid demo diagram of low-energy-consumption smart vehicle air-conditioning controller.

The most outstanding feature of this study is to build a low-energy-consumption smart air-conditioning control system (Figure 5) [24] without modifying the devices of the vehicle air-conditioning system. The system can detect vehicle's heat load, and control center follows vehicle's heat load to control the refrigerating capacity output from the air-conditioning system; when in-car environment meets the set temperature, it stops compressor's run at suitable timing; it has in-car space maintaining air temperature within the range that comforts human body, it reduces vehicle's oil consumption, which meets both comfort and environmental protection requirements. Solid demo diagram of the controller used in this study is graphically shown in Figure 6.

In this study, we first explore the overall heat load of vehicle. Heat load means all in-car heat sources, including conduction heat load from vehicle body, solar radiation heat load, latent and sensible heat load from out-of-car air, human body heat load, and vehicle power-equipment heat load. All heat loads can be detected by the outer environment temperature sensor and return air temperature sensor in the sensing unit, being sent back to CPU for processing and obtain the total heat load of vehicle.

After obtaining total heat load, when initiating the vehicle air-conditioning system, CPU receives the set target-temperature signal from set unit simultaneously; CPU calculates through the heat load demand and temperature setting and gets the air-conditioning capacity to be supplied by air-conditioning system and immediately decides compressor rpm and controls the refrigerant flow rate. Meanwhile, it changes fan speed per the heat-absorbing amount of evaporator and keeps the air-conditioning system running at optima operating efficiency status with minimum energy consumption.

### 2.3. Calculation of Vehicle Air-Conditioning Heat Load

We study the vehicle air-conditioning heat loads from in-car and out-of-car cases. In-car heat loads mainly come from human body and inner equipment; out-of-car loads include conduction heat load from vehicle body, solar radiation heat load, and latent and sensible heat load from out-of-car warm air.

#### 2.3.1. Human Body Heat Load

From ASHRAE 55a [26], human body heat load (*Q*_H_) can be divided into sensible heat and latent heat. From in-car passenger amount (*n*), human body heat load can be calculated from equations (1)–(3) [26].

The equation of human body sensible heat load is

(1) $$\begin{array}{c} Q_{\text{Hs}}=\;n\;\times \text{sensible heat load.} \end{array}$$

Sensible heat load of human body is 128.6 (W).

The equation of human body latent heat load is

(2) $$\begin{array}{c} Q_{\text{Hl}}=n\;\times \text{latent heat load.} \end{array}$$

Latent heat load of human body is 58.1 (W).

The equation of total human body heat load is

(3) $$\begin{array}{c} Q_{\text{H}}=Q_{\text{Hs}}+Q_{\text{Hl}}. \end{array}$$

#### 2.3.2. Conduction of Heat Load from Vehicle Body

Temperature difference between in-car and out-of-car exists; outer environmental heat can flow into in-car or out-of-car environment, and we have to consider the influence of heat conduction that affects in-car comfort. Vehicle-body heat-conduction ways can be divided to sheet metal and glass. According to ASHRAE 90.1 Specification [27], equation (4) displays heat conduction calculation equation [27].

*Q*_C_ is the vehicle-body conduction heat load, *u* is the thermal conductivity of heat-conducting material, *a* is the heat conduction area, and Δ*T* is the heat-conduction temperature difference:

(4) $$\begin{array}{c} Q_{\text{C}}=u\times a\times \Delta T. \end{array}$$

#### 2.3.3. Latent and Sensible Heat Load from Out-of-Car Warm Air

From ASHARE 62 Specification [28], each in-car person needs 2.5 L/s fresh air since ventilation will introduce fresh air and thus brings heat to in-car space. The calculation of outer air heat load can also be divided to latent and sensible heat load. Formula (5)–(7) display the calculation equations [28].

*Q*_O_ is the outer air heat load, *V* is the ventilation rate, *Q*_P_ is the steam latent, *C*_p_ is the air specific heat capacity, Δ*T* is the in-car/out-of-car temperature difference, and Δ*C* is the in-car/out-of-car humidity difference.

The equation of outer fresh air sensible heat load calculation is as follows:

(5) $$\begin{array}{c} Q_{\text{Os}}=V\times C_{\text{P}}\times \Delta T. \end{array}$$

The equation of outer fresh air latent heat load calculation is as follows:

(6) $$\begin{array}{c} Q_{\text{Ol}}=V\times C_{\text{p}}\times \Delta C. \end{array}$$

The equation of total outer fresh air heat load calculation is as follows:

(7) $$\begin{array}{c} Q_{\text{O}}=Q_{\text{Os}}+Q_{\text{Ol}}. \end{array}$$

#### 2.3.4. Solar Radiation Heat Load

From ASHRAE 90.1 Specification [27], vehicle is exposed to sunlight; heat of radiation will penetrate through glass windows; yet, part of radiate light is reflected by glass, part of it directly penetrates in and is absorbed by in-car articles and human body. Therefore, solar radiation is also the key influential factor of air-conditioning load. Equation (8) displays the calculation equation of radiation heat load [27].

*Q*_Sr_ is the radiation heat load, *Q*_*γ*_ is the solar radiation strength, *τ* is the transmittance, *a* is the glass area, *X* is the inner product in light direction and glass normal direction, and *Q*_d_ is the diffused light radiation strength:

(8) $$\begin{array}{c} Q_{\text{Sr}}=Q_{\gamma }\times \;\tau \;\times a\times X+Q_{\text{d}}\times \;\tau \;\times a. \end{array}$$

Vehicle's total heat load (*Q*_T_) mainly is the sum of human body heat load, vehicle-body conduction heat load, outer fresh air heat load, and solar radiation heat load. Equation (9) displays the calculation of total vehicle heat load (9):

(9) $$\begin{array}{c} Q_{\text{T}}=Q_{\text{H}}+Q_{\text{C}}+Q_{\text{O}}+Q_{\text{Sr}}. \end{array}$$

## 3. Experiment Arrangement and Tests of Low-Energy-Consumption Smart Vehicle AirConditioning Control System

As described above, in this study, we bring up the planning and design of low-energy-consumption smart vehicle air-conditioning control system structure and follow ASHRAE Specification to perform theoretic calculation of vehicle air-conditioning heat load. In this section, we give detailed description on the road-measurement plan of the studied vehicle air-conditioning system and provide performance verifying and efficiency comparison statements.

### 3.1. Road Measurement on Vehicle Air-Conditioning Control System

In this experiment, we totally selected three models of cars making tests; car models under tests are listed in Table 1.

The experimental specification and procedure of low-energy-consumption smart vehicle air-conditioning system are specified below:

1. Refrigerating capacity in the cars under test is certified; when in-car temperature is lower than 28°C, cars are run at idling speed; temperature at cooled air outlet shall be below 10 within 10 minutes.
2. Each car takes two tests: one was the car which was equipped with the in-car low-energy-consumption smart vehicle air-conditioning system; the other one was the car which used the original in-car air-conditioning control system. Both tests were performed at the same start point on road.
3. Drove the car on freeway at 110 km/hr ± 5 km/hr.
4. In the test that the car was equipped with the in-car low-energy-consumption smart vehicle air-conditioning system, the car was refueled to full, controlled temperature at 25°C by the controller making test; A/C wind speed s adjusted to maximum. After test run was over, we went back to gas station, refueled to full by autostops for three times, and recorded the km driven and refuel amount.
5. For the test of using original in-car air-conditioning control, we performed the test with the procedure same as the above one. We applied the original vehicle air-conditioning control mode; A/C wind speed was adjusted to maximum and after test run was over, we went back to gas station, refueled to full by autostops for three times, and recorded the mileage driven and refuel amount.
6. During the test runs, we recorded the max/min in-car temperature/humidity.

After the test cars run at different control modes, we recorded the mileage driven and refuel amount; equation (10) is followed to calculate the fuel-save percentage; in-car temperature/humidity was recorded by instruments listed in Table 2. The suggested comfort range specified in ASHRAE 55 is referred [29]. In Taiwan, comfortable temperature is 23°C∼26°C in summer and 18°C∼20°C in winter and humidity is within 50%∼60% RH.

The equation calculating fuel-save percentage:

(10) $$\begin{array}{c} \text{Fuel}-\text{save rate}\left(\%\right)=\frac{S_{\text{i}}-S_{\text{f}}}{S_{\text{f}}}\times 100\%. \end{array}$$

where *S*_i_: system-installed specific fuel consumption and *S*_f_: system-free specific fuel consumption.

### 3.2. Performance Verification and Efficiency Comparison

From the oil consumptions at the statuses of car using and not using the low-energy-consumption smart vehicle air-conditioning control system, whether or not controlling compressor can reduce how many oil consumption is realized.  Table 3 shows the outer environmental parameters and vehicle mileage on the road-test day. From Table 4, we can see that cars equipped with the smart control system presented in this study can adjust the output of compressor capacity according to the in-car heat load, sustained the in-car space within the range of comfortable temperature. The compressor rested for 16∼23 times and the smart control system effectively reduced the compressor load and obtained fuel-save target; fuel-save rate was 21%∼28% and, in particular, was effective to cars with lower emission capacity.

**Figure 7:**
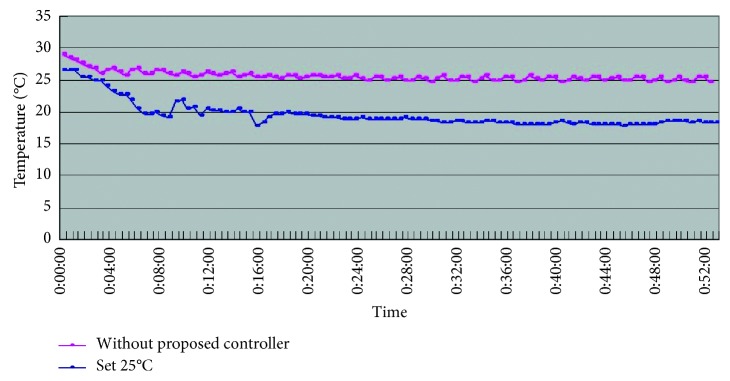
Car A in-car temperature change curve.

**Figure 8:**
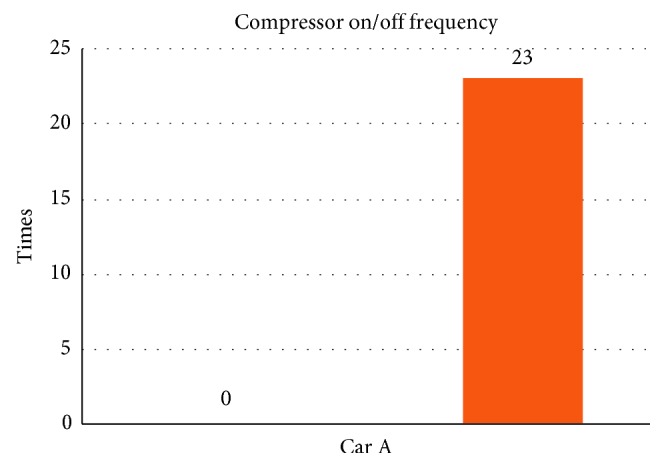
Air-conditioning compressor rest times for Car A.

**Figure 9:**
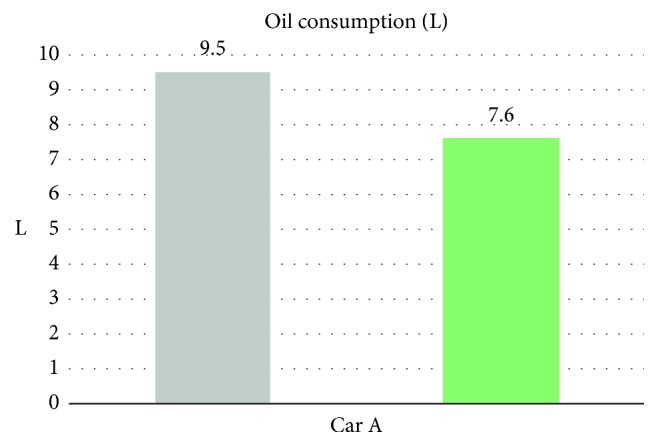
Oil consumption for Car A.

**Figure 10:**
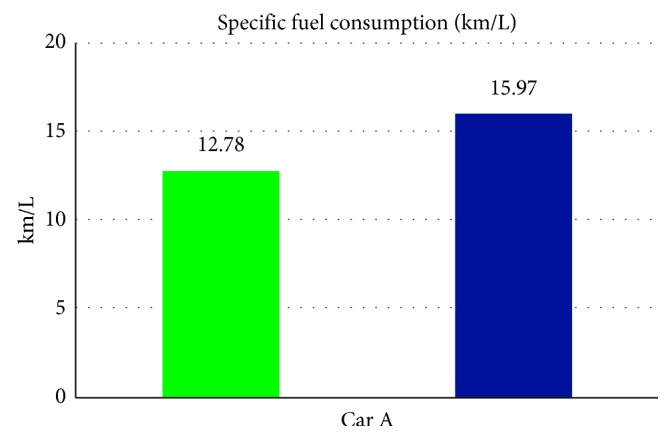
Specific fuel consumption for Car A.

From Table 5 we can see that, while being without the smart control system, when in-car heat load approached stability, Car A (Matrix) in-car temperature fluctuated within 16.8°C∼20.2°C, and temperature deviation reached 3.4°C and average temperature was 18.8°C; RH fluctuated within 32.3%∼38.9%, average humidity was 34.6%; in-car environment presented a low temperature/humidity status, lower than the suggested comfortable range. However, with the smart control system, when in-car heat load approached stability, the in-car temperature fluctuated within 24.7°C∼25.5°C, temperature deviation was only 0.8°C, average temperature sustained at 25.2°C; RH fluctuated within 48.7%∼58.1%, and average humidity was 54.4%; in-car average temperature sustained close to the set one, making in-car driver and passengers feel comfortable. From Figure 7 we can see that the experiment result shows that while without the smart control system, in-car temperature was 20°C, which was too low; the temperature deviation amplitude also was too big. However, with the smart control system, in-car temperature sustained at the set target point (25°C) and temperature deviation was very tiny. From Figure 8, we can see the experiment result showing that while without the proposed smart control system and when the temperature inside the cabin reaches the preset temperature, the air-conditioning compressor continues to work without rest. As a result, the oil consumption is 9.5 L, as shown in Figure 9. The specific fuel consumption is about 12.78 km/L, as shown in Figure 10. Compared with the test vehicle with the proposed smart control system, we see the temperature inside the cabin is controlled at the preset temperature and the air-conditioning compressor breaks 23 times during the evaluation period (one hour), as shown in Figure 8. As a result, the oil consumption is 7.6 L, as shown in Figure 9, saving 1.9 L of fuel consumption. In addition, the specific fuel consumption is about 15.97 km/L, as shown in Figure 10, and its fuel economy is about 25%.

**Figure 11:**
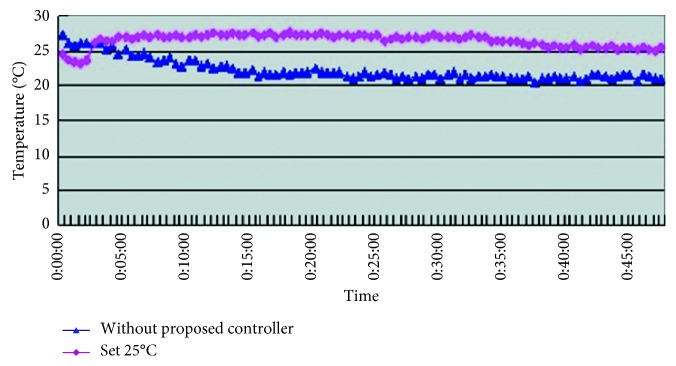
Car B in-car temperature change curve.

**Figure 12:**
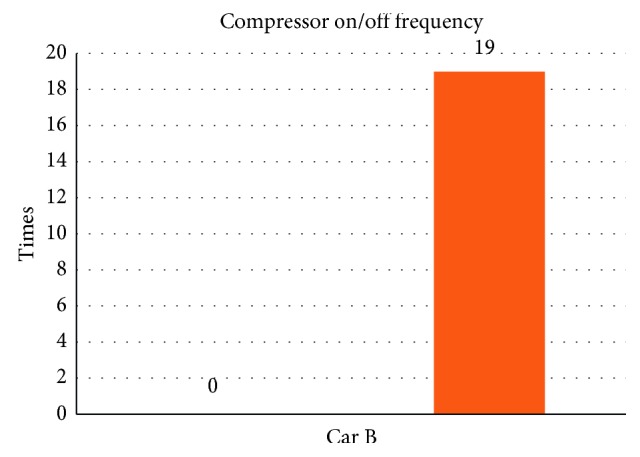
Air-conditioning compressor rest times for car B.

**Figure 13:**
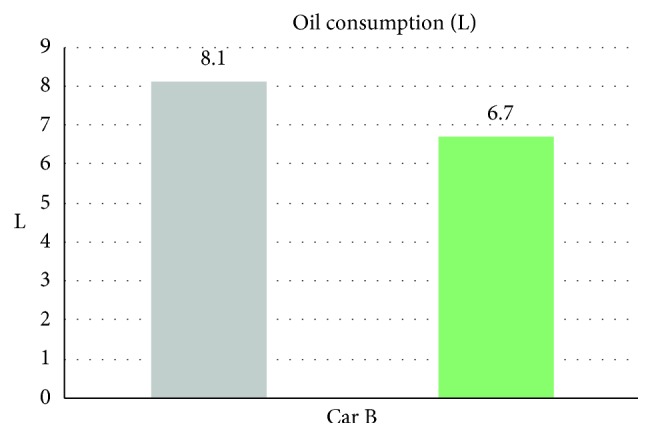
Oil consumption for car B.

**Figure 14:**
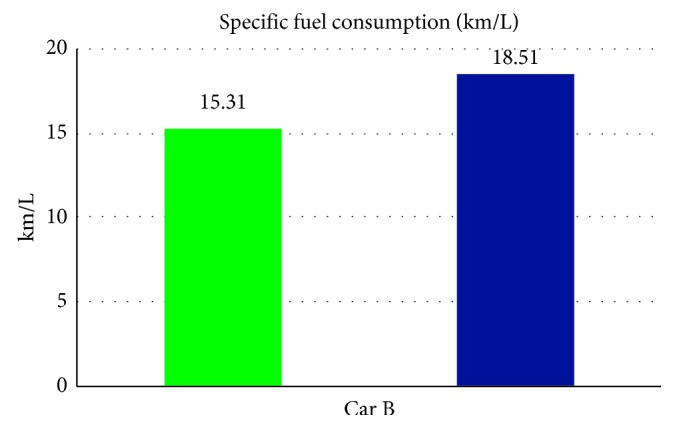
Specific fuel consumption for car B.

From Table 6 we can see that, while without the smart control system, when in-car heat load approached stability, Car B (Lancer) in-car temperature fluctuated within 20.4°C∼25.2°C, temperature deviation reached 4.8°C, and average temperature was 22.2°C; RH fluctuated within 33.3%∼36.7% and average humidity was 35.3%; in-car environment presented a low temperature/humidity status, lower than the suggested comfortable range. While with the smart control system, when in-car heat load approached stability, the in-car temperature fluctuated within 25.5°C∼27.6°C, temperature deviation was only 2.1°C, average temperature sustained at 26.2°C; RH fluctuated within 41.5%∼56.2%, and average humidity was 50.1%; in-car average temperature was slightly higher than the set one, but still was within the allowable range, making in-car driver and passengers feel comfortable. From Figure 11 we can see, the experiment result shows that while without the smart control system, in-car temperature sustained within 20°C∼22°C, which was too low; the temperature deviation amplitude also was too big. While with the smart control system, after certain time, in-car temperature became stable, sustained at the set target point (25°C), temperature deviation was very tiny. From Figure 12 we can see the experiment result shows that while without the proposed smart control system and when the temperature inside the cabin reaches the preset temperature, the air-conditioning compressor continues to work without rest. As a result, the oil consumption is 8.1 L, as shown in Figure 13, and the specific fuel consumption is about 15.31 km/L, as shown in Figure 14. Compared with the test vehicle with the proposed smart control system, we see the temperature inside the cabin is controlled at the preset temperature and the number of air-conditioning compressor breaks is 19 times during the evaluation period (one hour), as shown in Figure 12. As a result, the oil consumption is 6.7 L, as shown in Figure 13, saving 1.4 L of fuel consumption. The specific fuel consumption is about 18.51 km/L, as shown in Figure 14, and its fuel economy is about 21%.

**Figure 15:**
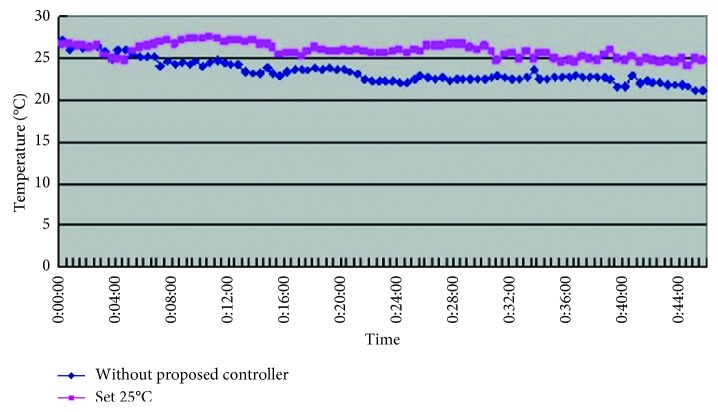
Car C in-car temperature change curve.

**Figure 16:**
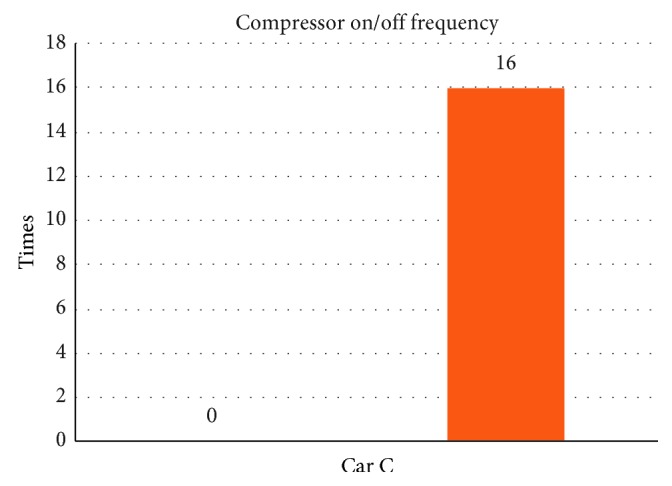
Air-conditioning compressor rest times for Car C.

**Figure 17:**
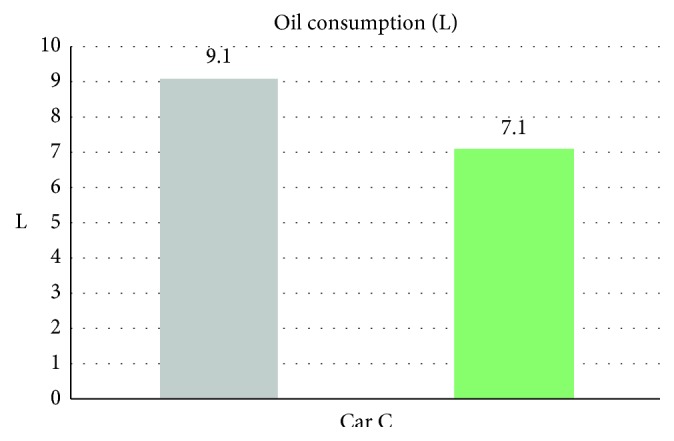
Oil consumption for Car C.

**Figure 18:**
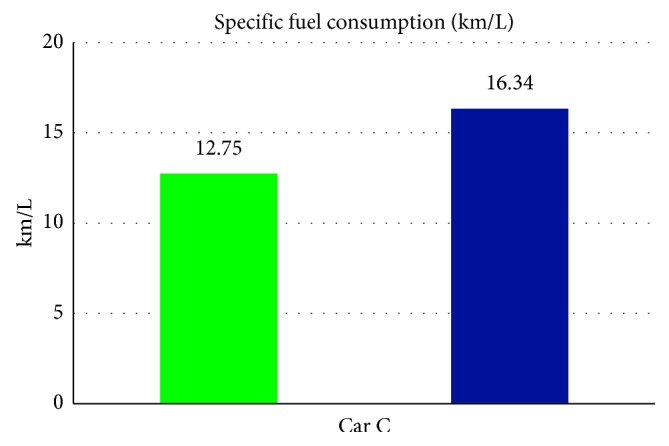
Specific fuel consumption for Car C.

From Table 7 we can see that, while without the smart control system, when in-car heat load approached stability, Car C (Solio) in-car temperature fluctuated within 21.8°C∼25.3°C, temperature deviation reached 3.5°C, average temperature was 23.4°C, RH fluctuated within 30.6%∼37.8%, and average humidity was 33.4% and in-car environment presented a low temperature/humidity status, lower than the suggested comfortable range. However, with the smart control system, when in-car heat load approached stability, the in-car temperature fluctuated within 24.1°C∼27.4°C; temperature deviation was only 3.3°C, average temperature sustained at 25.5°C; RH fluctuated within 43%∼52.8%, average humidity was 46.6%; in-car average temperature was slightly higher than the set one but still was within the allowable range, making in-car driver and passengers feel comfortable. From Figure 15 we can see, the experiment result shows that while without the smart control system, in-car temperature continued to go down from approx. 26°C to close to 20°C, making the in-car environment be in too-low temperature state. While with the smart control system, after certain time, in-car temperature became stable, at the set target point (25°C) is sustained. Since Car C emission capacity is lower than Car A and Car B, the temperature deviation amplitude was bigger than Car A and Car B. From Figure 16, we can see the experiment result shows that while without the proposed smart control system and when the temperature inside the cabin reaches the set temperature, the air-conditioning compressor continues to work without rest. As a result, the oil consumption is 9.1 L, as shown in Figure 17, and the specific fuel consumption is about 12.75 km/L, as shown in Figure 18. Compared with the test vehicle with the proposed smart control system, we see the temperature inside the cabin is controlled at the preset temperature, the number of air-conditioning compressor breaks is 23 times during the evaluation period (one hour), as shown in Figure 16. As a result, the oil consumption is 7.1 L, as shown in Figure 17, saving 2 L of fuel consumption. The specific fuel consumption is about 16.34 km/L, as shown in Figure 18, and its fuel economy is about 28%.

From the experiment, we can see that the low-energy-consumption smart vehicle air-conditioning control system revealed from this study can solve the problems of low in-car air humidity and significant oil consumption rate consumed by the air-conditioning system, occurred in conventional vehicle air-conditioning system that uses mixing of warm and cold air to regulate in-car environmental temperature. The control system introduced in this study does not need to modify vehicle air-conditioning system devices; what it needs to do is merely add the study-introduced new low-energy-consumption smart vehicle air-conditioning control system. The system thus becomes able to detect the total heat load, change the air-conditioning capacity mode of air-conditioning system according to the heat load detected, sustain in-car environment comfortable, and implement the idea of supplying refrigerating capacity to balance heat load accurately. When in-car environment reaches the target temperature, the system will properly halt compressor to have in-car space maintain in a comfortable temperature range; and be able to reduce vehicle's oil consumption. Comparison of the features of studied specific low-energy-consumption smart vehicle air-conditioning control system against the conventional air-conditioning system is shown in Table 8.

This study is limited to the fact that the vehicles under test are mainly based on traditional fuel engine. From the above experiments, we can conclude that we can obtain effective control for the comfort level inside the cabin and achieve energy-saving benefits for traditional vehicles by using the proposed air-conditioning system. In the future, the air-conditioning system of the hybrid electric vehicle and the electric vehicle can also be combined with the intelligent control mode of the proposed system to improve the environmental comfort level and endurance of vehicle while achieving energy saving.

## 4. Conclusion

From the experimental result, we can see that the planned system can get significant benefit from vehicle air-conditioning thermostatic control; in-car average temperature can be maintained within 25.2°C∼26.2°C; when in-car heat load is stable, temperature fluctuating range is tiny; in-car average humidity can be maintained within 46.6%∼54.4%; both parameters are within the suggested range, enabling in-car passengers to enjoy the comfortable environment. When maintaining in-car environment at a comfortable temperature range, the halting of compressor can reach 16∼23 times and can save fuel; fuel-saving percentage reaches 21%∼28%. From the experimental result, we get the conclusion: low emission-capacity vehicles can get better fuel-saving effect. The reason why we bring up the low-energy-consumption smart vehicle air-conditioning control system in this study is to offer a thermostatic in-car environment to the driver and passengers. While the low-energy-consumption air-conditioning control system is working, the system can calculate the total in-car heat load and provide equivalent refrigerating capacity to balance it. This function can effectively reduce compressor load and acquire minimum vehicle oil consumption when the vehicle air-conditioning system is running. The other feature of the smart vehicle air-conditioning control system shown in this study is that it needs not to modify vehicle air-conditioning system devices; just add the Study-introduced new low-energy-consumption smart vehicle air-conditioning control system. It can provide comfortable in-car space to the driver and passengers and reduce vehicle fuel consumption, slow down the speed of running out the planet's energy, and improve the environmental pollution condition.

## Figures and Tables

**Table 1:** Car models under tests.

No.	Models	Engine displacement
1	A	1600 CC
2	B	1600 CC
3	C	1300 CC

**Table 2:** Measuring instruments specification table.

Brand	Model	Detection range
TES	TES-1370 NDIR CO_2_ meter	CO_2_: 0∼6000 ppm, diffusion sampling
		Humidity: 10%∼95% RH
		Temperature: −20°C∼+60°C/−4℉∼+140℉

**Table 3:** Outer environment parameters.

Models	Route	Set temperature	Average temperature (°C)	Average humidity (%)
A	Highway	Without proposed controller	30.5	51.8
		25°C	32.4	58.8
B	Highway	Without proposed controller	31.8	74.2
		25°C	34.5	63.5
C	Highway	Without proposed controller	32.5	63.6
		25°C	33.8	58

**Table 4:** Actual fuel consumptions of cars at road test.

Models	Route	Set temperature	Compressor on/off frequency	Mileage (km)	Oil consumption (L)	Specific fuel consumption (km/L)	Fuel economy (%)
A	Highway	Without proposed controller	0	121.4	9.5	12.78	25
		25°C	23	121.4	7.6	15.97	
B	Highway	Without proposed controller	0	122	8.1	15.31	21
		25°C	19	122	6.7	18.51	
C	Highway	Without proposed controller	0	120.8	9.1	12.75	28
		25°C	16	120.8	7.1	16.34	

**Table 5:** Car A in-car temperature/humidity change table.

Route		Highway	
Set temperature		Without proposed controller	25°C
Temperature (°C)	Range	16.8°C∼20.2°C	24.7°C∼25.5°C
	Average	18.8°C	25.2°C

Humidity (%)	Range	32.3%∼38.9%	48.7%∼58.1%
	Average	34.6%	54.4%

**Table 6:** Car B in-car temperature/humidity change table.

Route		Highway	
Set temperature		Without proposed controller	25°C
Temperature (°C)	Range	20.4°C∼25.3°C	25.5°C∼27.6°C
	Average	22.2°C	26.2°C

Humidity (%)	Range	33.3%∼36.7%	41.5%∼56.2%
	Average	35.3%	50.1%

**Table 7:** Car C in-car temperature/humidity change table.

Route		Highway	
Set temperature		Without proposed controller	25°C
Temperature (°C)	Range	21.8°C∼25.3°C	24.1°C∼27.4°C
	Average	23.4°C	25.5°C

Humidity (%)	Range	30.6%∼37.8%	43%∼52.8%
	Average	33.4%	46.6%

**Table 8:** Comparison table of low-energy-consumption smart vehicle air-conditioning control system vs. conventional air-conditioning system.

Item	This subjected system	Ordinary vehicle air-conditioning system
Thermostatic control	Smart control system	Air-mixing box
Air-conditioning capacity	Variable energy volume	Affect engine power output
Average temperature (°C)	25.2∼26.2	18.8∼23.4
Average humidity (%)	46.6∼54.4	33.4∼35.3
Temp. deviation	Small	Large
Grade of comfort	Sustain envir., temp., and RH	Low temp. and RH
Specific fuel consumption (km/L)	15.97∼18.51	12.78∼15.31
Oil consumption (L)	6.7∼7.6	8.1∼9.5

## Data Availability

The parameters and test data regarding this research are available on the following website. Please visit the following: https://drive.google.com/file/d/1KNHzyBGkgMGjZqHxdwnnI9WUH61MT5EY/view?usp=sharing.
